# Late Bladder Rupture and Strangulated Ileus Following Radical Hysterectomy and Radiation Therapy for Cervical Cancer

**DOI:** 10.7759/cureus.81016

**Published:** 2025-03-22

**Authors:** Chika Morita, Yusuke Fujii, Saki Maemura, Takashi Yasuda, Keitaro Kakinoki

**Affiliations:** 1 Department of General and Gastroenterological Surgery, Hyogo Prefectural Harima-Himeji General Medical Center, Himeji, JPN

**Keywords:** bladder rupture, cervical cancer, internal hernia, radiation therapy, strangulated ileus

## Abstract

Bladder rupture can occur as a late complication of radiation therapy targeting the pelvic organs. It is often diagnosed as peritonitis caused by intraperitoneal urine leakage. We report a case of an internal hernia and strangulated ileus caused by a bladder wall defect that occurred due to bladder rupture, which served as the hernia orifice.

The patient was a woman in her 70s who had undergone radical hysterectomy and postoperative radiation therapy for cervical cancer 37 years prior. She was diagnosed with a strangulated ileus and underwent emergency surgery for abdominal pain. Intraoperative findings revealed moderate amounts of clear yellow ascites and a bladder wall defect with small intestinal incarceration, leading to a strangulated ileus. The strangulated bowel was resected and the bladder wall was trimmed and sutured. A histological examination of the trimmed bladder wall revealed findings consistent with late complications of radiation cystitis.

Bladder rupture is characterized by nonspecific findings, making the diagnosis difficult. It is often diagnosed during surgery for acute abdomen. In this case, the small intestine was incarcerated at the bladder rupture site, causing strangulated ileus, a previously unreported condition. Although rare, bladder rupture should be considered a cause of strangulated ileus in the pelvic region.

## Introduction

Bladder rupture can be classified as traumatic or non-traumatic (spontaneous) based on its cause, with traumatic cases being more common. Spontaneous rupture can be caused mainly by bladder wall fragility or bladder overdistension. Bladder wall fragility can be caused by cystitis, bladder cancer, and radiation therapy, while bladder overdistension can be caused by conditions such as prostatic hypertrophy, heavy alcohol consumption, or neurogenic bladder [1]. Reports of spontaneous bladder rupture caused by urinary retention due to alcohol consumption were common in the past, but recent reports have increased regarding neurogenic bladder after hysterectomy and radiation cystitis after radiation therapy [2].

With pelvic radiation therapy, radiation cystitis, including bladder wall inflammation and ulceration, develops in 5-10% of patients [3]. The radiation tissues include fibrosis of the submucosal tissue and atrophy, degenerative necrosis, and desquamation of the epithelium due to vascular occlusion. This results in reduced compliance of the bladder wall and delayed wound healing [4].　

Bladder rupture is often diagnosed based on symptoms of peritonitis caused by intraperitoneal urine leakage [2]. The typical symptoms include sudden severe abdominal pain, hematuria, and difficulty urinating [5]. Diagnosis is confirmed through imaging techniques such as computed tomography (CT) scans, cystoscope, or cystography, which can reveal the extent and location of the rupture, but often it remains unrecognized until revealed intraoperatively [6,7]. Sudden abdominal pain and ascites accumulation on CT may indicate an acute abdomen such as strangulated ileus or perforation of the gastrointestinal tract. Thus, an emergency physician or surgeon is often responsible for the initial diagnosis, but preoperative diagnosis is difficult.

Treatment options vary depending on the severity of the rupture, ranging from conservative management with urinary catheterization to surgical repair [5]. Post-treatment follow-up is crucial to monitor for potential complications and ensure proper healing.

We encountered a case of an internal hernia and strangulated ileus caused by a bladder wall defect due to late complications of radiation therapy for cervical cancer. This condition, in which the bladder wall defect serves as the hernia orifice, has not been reported previously. However, with advancements in multimodal therapy, including radiation therapy for pelvic malignancies, and improved long-term survival, we might see similar cases.

## Case presentation

A 78-year-old woman presented with abdominal pain that had persisted for 10 days. She had a history of radical hysterectomy and postoperative radiation therapy for cervical cancer 37 years prior, with subsequent urinary retention managed by intermittent self-catheterization. The details of the postoperative radiation therapy are unknown.

Upon presentation, her vital signs were stable (blood pressure: 129/74 mmHg; pulse: 62 bpm), and she had mild abdominal distension with tenderness in the left lower abdomen, but no peritoneal signs. Blood tests revealed mild anemia, renal impairment, and slightly elevated C-reactive protein (CRP) levels (Table 1). A urinalysis revealed pyuria and hematuria (both 3+). A mildly elevated inflammation may be associated with a urinary tract infection or nonspecific elevated inflammatory response at the time of bladder rupture. It is not clear whether the renal function is due to ileus or pseudo-renal failure. Pseudo-renal failure is a "reverse auto-dialysis" condition in which blood urea nitrogen and creatinine are reabsorbed via the peritoneum due to the influx of urine into the peritoneal cavity. The renal function may be normal, but the condition is the same as renal impairment [8].

**Table 1:** Blood tests Mildly elevated inflammation is due to a urinary tract infection or nonspecific reaction. It is not clear whether renal dysfunction is due to ileus or pseudo-renal failure. WBC: white blood cell; Hb: hemoglobin; CRP: C-reactive protein; BUN: blood urea nitrogen; Cr: creatinine

Parameters	Patient values	Reference range
WBC	5.4×10³/µL	3.3-8.6×10³/µL
Hb	10.1 g/dL	11.6-14.8 g/dL
CRP	1.44 mg/dL	0.00-0.14 mg/dL
BUN	31.6 mg/dL	8.0-20.0 mg/dL
Cr	0.91 mg/dL	0.46-0.79 mg/dL

**Figure 1:**
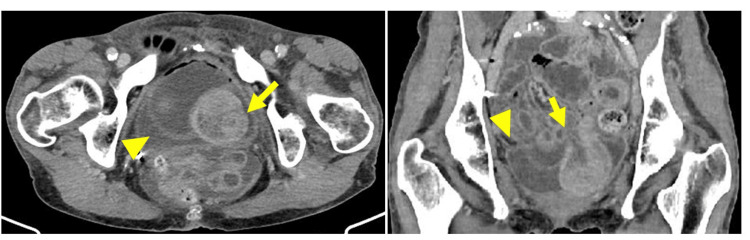
Enhanced CT A: Horizontal: the arrow indicates the small intestine forming loops. The arrowhead points to the bladder; the continuity of the bladder wall appears to be broken. In the horizontal section, it is not clear whether the small intestine is inserted into the bladder. B: Coronal: the small intestine at the arrow forms a closed loop (arrow). The mouth side of the intestine is dilated from there, and it is diagnosed as strangulated ileus. The arrowhead indicates the bladder. Retrospectively, the small intestine is stuck in the ruptured bladder on CT, as seen in the surgical findings. However, it would be difficult to obtain a definitive diagnosis without bladder rupture in mind. CT: computed tomography

Contrast-enhanced CT revealed moderate ascites and a closed loop of the small intestine near the bladder (Fig. 1A, 1B). Bladder rupture was not in the differential at this time. Based on the symptoms of abdominal pain and imaging findings with closed loop and ascites, she was diagnosed with an internal hernia and strangulated ileus due to adhesions from the previous surgery. Release of the internal hernia is considered necessary; she underwent emergency surgery. Since the patient's vital signs were stable, the surgery was first started by laparoscopy, which is minimally invasive.

**Figure 2:**
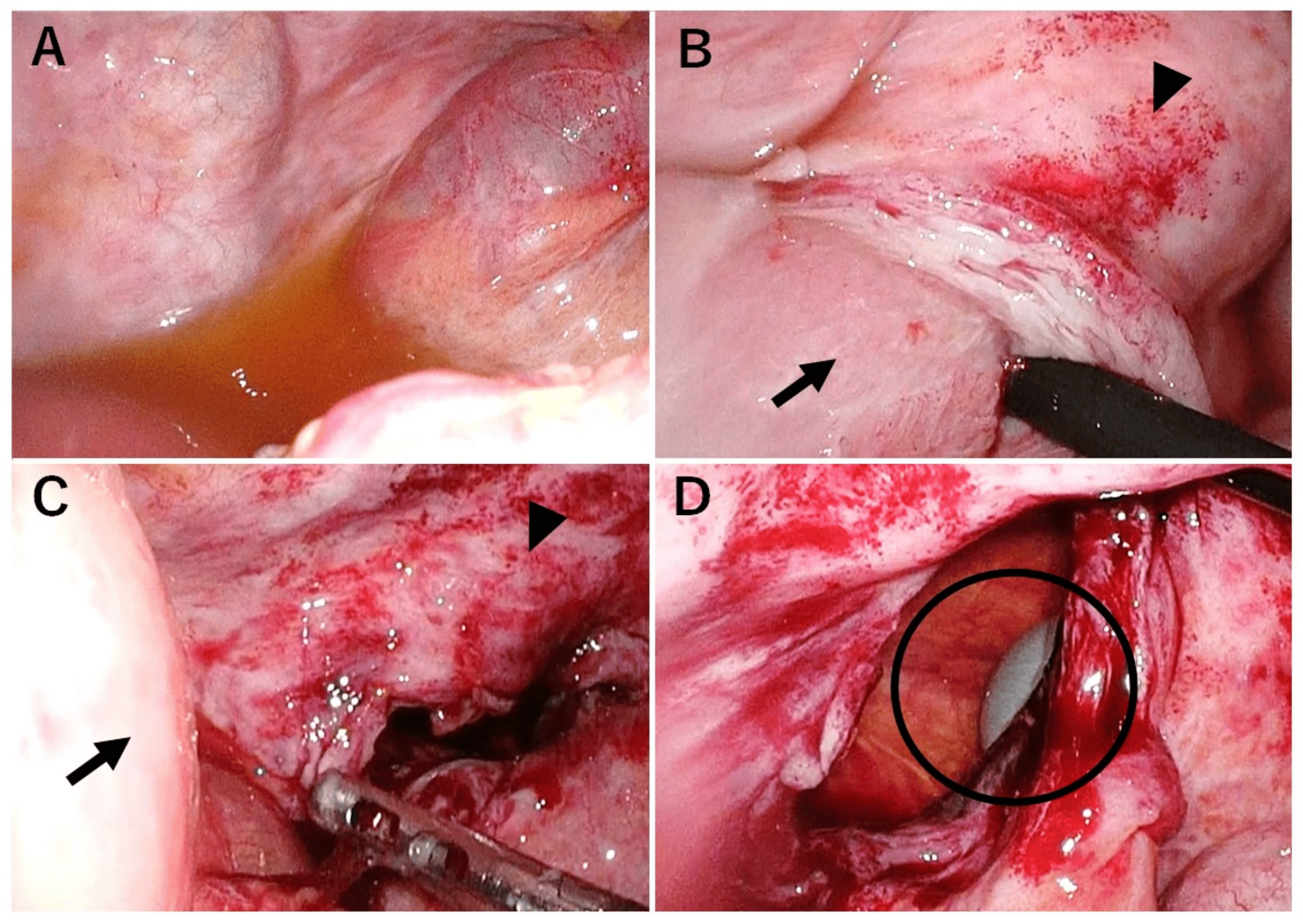
Laparoscopic surgery findings A: A small amount of pale-yellow transparent ascites on the pelvic floor. B: The small intestine (arrow) is detached from the adhesions to the bladder (arrowhead). We thought that the adhesions of the small intestine and bladder lead to ileus release. C: The small intestine with unsettling (arrow) and the bladder (arrowhead). D: The bladder catheter was exposed (circle), and the small intestine was found inserted into the bladder lumen. This finding revealed for the first time that a ruptured bladder was the cause and the small intestine was fitted into the bladder.

Intraoperative findings included moderate amounts of clear yellow ascites and incarceration of the small intestine in the bladder wall, with firm adhesion (Fig. 2A, 2B). Since the preoperative CT showed a lesion near the bladder, the first step was dissection of the adhesion between the small intestine and the bladder. Thereupon, the incarcerated small intestine was released, and the bladder wall defect was identified with a urinary catheter visible through the defect, indicating a bladder rupture (Fig. 2C, 2D). 

**Figure 3:**
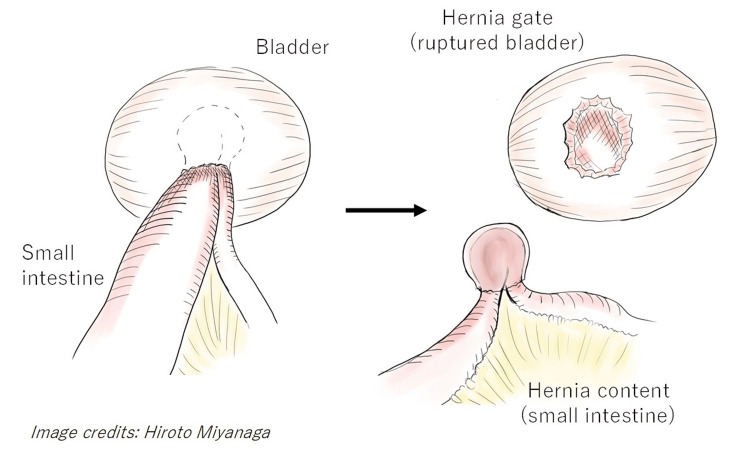
Schematic illustration of surgical findings The ruptured bladder herniated with the small intestine as hernia contents.

It was only at this point that the small intestine was fitted into the bladder, causing the strangulated ileus to become clear (Fig. 3). 

**Figure 4:**
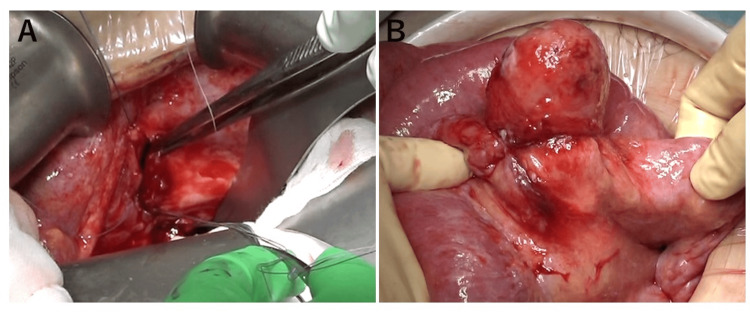
Open abdominal surgery findings A: The area around the ruptured bladder was trimmed for pathological examination. The bladder was then sutured closed. B: The aborted small intestine was partially resected and submitted to pathological examination.

The bladder wall was trimmed and sutured. The strangulated bowel was resected because it may become stenotic (Fig. 4A, 4B). The surgery lasted for two hours and 53 minutes, with minimal blood loss. An analysis of the ascitic fluid revealed elevated creatinine levels, indicating urine leakage from a bladder rupture.

**Figure 5:**
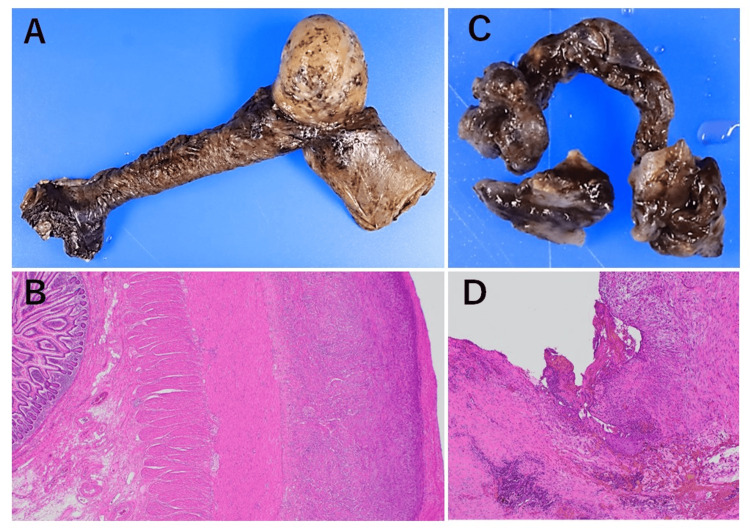
Pathological findings A, B: The small intestine serosa showed fibrin deposition, increased granulation tissue, and neutrophilic infiltrate. C, D: Ruptured bladder. The mucosa showed erosions, no muscle layer, serous membrane fibrosis, and fragility, which imply the effects of irradiation.

Postoperatively, the patient had a permanent urinary catheter due to persistent urinary retention, but otherwise recovered well and was discharged on postoperative day 23. Histological examination of the trimmed bladder wall showed fibrosis and fragility consistent with radiation cystitis. This finding suggests that the cause of bladder rupture is consistent with bladder wall weakening due to radiation cystitis. The resected small intestine showed severe peritonitis with fibrin deposition and neutrophil infiltration (Fig. 5A-5D). No malignancy was observed in any of the tissues.

## Discussion

Bladder rupture is relatively rare and still difficult to diagnose if not listed as a differential disease. Symptoms often include abdominal pain due to peritonitis caused by urine leakage, with hematuria and urinary retention being less common. Anatomically, the bladder dome is more prone to rupture due to its lack of surrounding supportive tissue and thinner detrusor muscle than others, so intraperitoneal rupture is more common than extraperitoneal rupture [9]. Imaging may reveal ascites and bowel distension due to urine leakage, making differentiation from gastrointestinal perforation or obstruction difficult.

Cystoscopy and cystography are useful for a definitive diagnosis [7]. Urologists perform cystoscopy and other procedures in anticipation of bladder rupture, so the diagnosis is more likely to be made. On the other hand, surgeons and emergency physicians have difficulty assuming bladder rupture from CT. Most often, there is ascites due to urinary overflow or intestinal dilatation due to peritonitis. Occasionally, there are cases with air in the abdominal cavity. Symptoms of abdominal pain and imaging studies often indicate strangulated ileus or perforation of the gastrointestinal tract, and emergency surgery is often performed. Also, cystoscopy is difficult to identify if the rupture is small, or cystourethrography may not confirm contrast overflow unless the bladder is well distended [7]. A combination of various modalities is useful for a definitive diagnosis.

Conservative treatment is an option for mild disease, but the wall itself is fragile or has a background condition that causes hyperextension of the wall, so recurrence is likely to occur [2]. Surgical repair is desirable, but because of the underlying weakness of the wall, recurrence is possible even after surgery. Bladder decompression procedures such as self-urination or bladder catheterization are also important.

The fragility of the bladder wall is caused by radiation damage. Early radiation cystitis involves inflammation, mucosal edema, and hyperemia of the bladder wall, whereas late radiation cystitis results in decreased bladder compliance and delayed wound healing [3]. Histologically, fibrosis of the submucosa and muscularis, vascular occlusion, epithelial atrophy, degeneration, necrosis, and detachment occur, leading to bladder rupture [4]. In radiation cystitis that leads to bladder rupture, there are severe degeneration and desquamation of the muscle layer. Radiation-induced bladder rupture commonly occurs in a long term greater than 10 years after radiation therapy [10]. It is important to make assumptions from the medical history interview.

Radiation cystitis is a factor in bladder rupture, but there are few reports of bladder rupture caused only by radiation therapy. In addition, most cases occur after surgery for uterine cancer although radiation cystitis can occur after radiation therapy for rectal, bladder, and prostate cancer. There have also been reports of bladder rupture following radiation therapy for prostate cancer [11], and nerve damage due to uterine cancer surgery is also a contributing factor. Long-term complications such as autonomic nerve damage can occur after radical hysterectomy, including sympathetic nerve damage which occurs in approximately 50% of patients [12]. Autonomic nerve injuries can lead to neurogenic bladder and increased bladder pressure, contributing to bladder rupture. Fujikawa et al. reported that the incidence of bladder rupture after radiation therapy for gynecologic cancers was 2.1%, not as rare as previously thought [13].

The primary treatment for microinvasive cervical carcinoma (International Federation of Gynecology and Obstetrics (FIGO) stage IA) is surgery. In contrast, radiation therapy can be chosen as one of the main treatments for invasive cervical carcinoma (FIGO stage IB or later) [14]. The five-year relative survival rate of patients with localized cervical cancer is 91.1%; the rate decreases to 60.8% if the disease spreads to nearby tissues, organs, or regional lymph nodes and 19.4% if it spreads to a distant part of the body [15]. Cervical cancer has become a malignancy with a high potential for a cure or long-term survival with appropriate treatment.

In the present case, details are unknown, but the patient underwent a radical hysterectomy and postoperative radiation therapy for cervical cancer. The preoperative diagnosis suggested strangulated ileus due to adhesions, but bladder rupture was not diagnosed until an intraoperative examination revealed bladder rupture and internal hernia with small intestinal incarceration. This is a rare and valuable case, highlighting the need to consider bladder rupture as a potential cause of strangulated ileus in patients with a history of pelvic radiation therapy or cervical cancer.

With advancements in multimodal therapy, including surgery, radiation therapy, and chemotherapy, the long-term survival after treatment for malignancies has improved. The aging of the population with improved long-term prognosis will very likely lead to increased dysuria and worsening of bladder wall fragility. Consequently, the incidence of bladder rupture, which has rarely been reported, may increase.

## Conclusions

Advances in multimodal therapy have significantly improved the prognosis of patients with pelvic malignancies. A better prognosis increases the age range of patients. Reports of bladder rupture are likely to increase in the future due to the aging population's increasing dysuria and worsening wall fragility over time.

This unique case underscores the need for vigilance regarding late-onset complications in patients with a history such as after uterine cancer surgery or pelvic radiation therapy.　

Our findings highlight the importance of early detection and intervention in such cases to prevent severe outcomes. Although this case is much rarer, clinicians should consider bladder rupture as a differential diagnosis in patients presenting with acute abdominal symptoms, especially those with a history of pelvic malignancies and radiation therapy. Further research is warranted to explore the mechanisms underlying radiation-induced bladder fragility and to develop preventive strategies.

## References

[1] Mitchell T, Al-Hayek S, Patel B, Court F, Gilbert H (2011). Acute abdomen caused by bladder rupture attributable to neurogenic bladder dysfunction following a stroke: a case report. J Med Case Rep.

[2] Hayashida H, Mabuchi S, Kawamura N, Matsuzaki S, Hisa T, Kamiura S (2023). Spontaneous bladder rupture attributable to a radical hysterectomy-associated neurogenic bladder in patients with cervical cancer: a case report and literature review. Int J Surg Case Rep.

[3] Lobo N, Kulkarni M, Hughes S, Nair R, Khan MS, Thurairaja R (2018). Urologic complications following pelvic radiotherapy. Urology.

[4] Suresh UR, Smith VJ, Lupton EW, Haboubi NY (1993). Radiation disease of the urinary tract: histological features of 18 cases. J Clin Pathol.

[5] Zhang Y, Yuan S, Alshayyah RW (2021). Spontaneous rupture of urinary bladder: two case reports and review of literature. Front Surg.

[6] Addar MH, Stuart GC, Nation JG, Shumsky AG (1996). Spontaneous rupture of the urinary bladder: a late complication of radiotherapy--case report and review of the literature. Gynecol Oncol.

[7] Watanabe Y, Yamazaki S, Yokoyama H (2021). A rare case of recurrent generalized peritonitis caused by spontaneous urinary bladder rupture after radiotherapy: a case report and literature review. Medicines (Basel).

[8] Matsumura M, Ando N, Kumabe A, Dhaliwal G (2015). Pseudo-renal failure: bladder rupture with urinary ascites. BMJ Case Rep.

[9] Evans RA, Reece RW, Smith MJ (1976). Idiopathic rupture of the bladder. J Urol.

[10] Welp A, Fields EC, Randall L, Brown FK, Sullivan SA (2020). Acute extraperitoneal spontaneous bladder rupture in cervical cancer patient undergoing chemoradiation: a case report and review of the literature. Gynecol Oncol Rep.

[11] Basiri A, Radfar MH (2008). Conservative management of early bladder rupture after postoperative radiotherapy for prostate cancer. Urol J.

[12] Yalla SV, Andriole GL (1984). Vesicourethral dysfunction following pelvic visceral ablative surgery. J Urol.

[13] Fujikawa K, Yamamichi F, Nonomura M, Soeda A, Takeuchi H (1999). Spontaneous rupture of the urinary bladder is not a rare complication of radiotherapy for cervical cancer: report of six cases. Gynecol Oncol.

[14] Bhatla N, Aoki D, Sharma DN, Sankaranarayanan R (2021). Cancer of the cervix uteri: 2021 update. Int J Gynaecol Obstet.

[15] (2025). Cancer stat facts: cervical cancer. https://seer.cancer.gov/statfacts/html/cervix.html.

